# What Do We Talk About When We Talk About Frailty?

**DOI:** 10.3389/fresc.2021.633961

**Published:** 2021-10-07

**Authors:** Alberto Falchetti, Paolo Capodaglio

**Affiliations:** ^1^Bone Unit, S Giuseppe Hospital, IRCCS Istituto Auxologico Italiano, Piancavallo, Italy; ^2^Endocrine and Metabolic Research Laboratory, Istituto Auxologico Italiano, Milan, Italy; ^3^Rehabilitation Unit and Research Laboratory in Biomechanics and Rehabilitation, S Giuseppe Hospital, IRCCS Istituto Auxologico Italiano, Piancavallo, Italy; ^4^Department of Surgical Sciences, Physical and Rehabilitation Medicine, University of Torino, Torino, Italy

**Keywords:** frailty, metabolic syndrome, sarcopenia, osteoporosis, disability, rehabilitation

Currently, there are two major conceptual frameworks for the term “frailty”: (a) physical frailty, capturing representative signs and symptoms (fatigue, low activity, weakness, weight loss, and slow gait) of community-dwelling older adults that are most vulnerable to adverse health outcomes; (b) deficit accumulation frailty, identifying the most vulnerable older adults through cumulative dysmetabolic, chronic comorbidities. The two frameworks are interconnected and have a common denominator, which is advanced *vulnerability*, a generally held term capturing the essence of age-related decline. Despite being a widely acknowledged concept in global health, its translation to the general population health and health service delivery, planning, and utilization is in factual terms often not thoroughly pursued.

It has been assessed that decreases in the mortality rates have largely plateaued and many individuals spend longer lives in poor health conditions, often struggling with socially driven conditions such as “chronic” drug use or abuse. The recent plague of COVID-19 infection has (re) confirmed that underlying, hidden, forms of frailty can unmask under critical conditions. These variable forms of frailty, although predominant in the older subjects, are not exclusive to this population. Single or coexisting clinical manifestations of human organs and systems (e.g., complicated diabetes, dysmetabolic, neurodegenerative, cardiovascular conditions, myopathies, obesity, sarcopenia, rheumatic diseases, hypogonadism, fragility fractures, and osteomalacia) can determine a variety of outcomes ranging from asymptomatic, paucisymptomatic up to lethal. Therefore, diagnosing one or more already acclaimed frailty conditions can be too simplistic and reductionist. Instead, to precociously identify those who in their lifetime are likely to develop a certain degree of frailty, both age- and non-age-related, appears a more comprehensive and useful task. This means going beyond the orthogeriatric concept of frailty or, in other terms, going from regional to global frailty.

Dysmobility syndrome summarizes a cluster of coexisting conditions, such as osteoporosis, sarcopenia, obesity (sarcopenic and not), as well as other fragility fracture risk factors, that determine a higher risk for falls and fractures (1), representing a typical age-related condition of vulnerability. Currently, it is well known that most of the fragility fractures occur in people with DXA-assessed bone mineral density values in the osteopenic area, probably because the quality of their bones is deteriorated or has never been adequately built since childhood to adolescence for various reasons. If those subjects should sustain falls and/or are also sarcopenic, their bone frailty can be unmasked. Sarcopenia is largely undiagnosed in clinical care, but it remains linked to substantial fracture and fall risk. Metabolic syndrome represents a cluster of not necessarily age-related conditions that occur together and determine a higher risk for cardiovascular heart diseases, type 2 diabetes, increased blood pressure, excess body fat around the waist, and mixed dyslipidemia. This is a seemingly distinct entity from the dysmobility syndrome. However, the common denominators of the two syndromes are impaired energy balance, either in terms of its generation or dissipation/transformation, and low-physical activity levels. In the recent past, several studies, especially in animal models, have suggested that the skeleton is not only a “simple” calcium and phosphate deposit, responsive to calciotropic hormones, rather an actor playing an important role in energy metabolism through hormonal networks, such as adipokines, insulin/insulin-like growth factor 1, and carboxylated/under carboxylated osteocalcin ratio pathways (2), together with other organs involved in the metabolic control. Osteoblasts, myocytes, and adipocytes have common cellular origins from the mesenchymal stem cells, and this makes the hypothesis that the skeleton has a role in energy metabolism unsurprising. A combination of low bone and muscle mass, poor skeletal quality, low-muscle function, and relatively high-fat mass, also together with other factors, could better screen the progression toward general and/or single-, multiregional frailty and disability. However, it is not always clear if this combination represents the starting or the arrival point or whether they are just an epiphenomenon of a generic frailty syndrome. Recognition of a progression toward a low-physical activity level status, not necessarily age-related, secondary to metabolic and/or dysmobility syndrome or their combination, unifying osteoporosis, sarcopenia, obesity and diabetes, and cardiovascular risk, brings the focus back to a holistic view in which the patient is seen as a whole, and not just to his/her bones, fat, or lean mass separately. However, this holistic approach remains virtual in many cases. In addition, focusing upon “intrinsic capacity” (3–5) for the identification of individuals who may benefit from interventions, as well as for clinical outcome measures, can reorient public health and clinical practice away from the disease-orientated approach toward a more effective person-centered approach based on the functional evaluation, which will identify earlier opportunities for intervention. This should overcome the traditional approach by different specialists with segmented and not necessarily communicating paths, often associated with fractioning of clinical information and communication gaps between the various specialists that ultimately lead to a disrupted continuum of care.

Given such limitations, we reckon that a circular path approach might be presented with more advantages. A common multidisciplinary diagnostic-clinical-therapeutic management of patients that allows findings from the endocrinologist, bone specialist, cardiologist, and rheumatologist to flow to the PMR specialist to address global function (bone, muscle, and fat mass) more effectively (6). The PMR specialist circulates responses of the patient to rehabilitation treatment back to the other colleagues to optimize pharmacological therapies, in line with the general principles of precision medicine. Such a circular and personalized approach could provide us with a larger therapeutic “armamentarium” for preventing functional decline and minimizing disability starting from the younger ages than in a traditional geriatric rehabilitation approach. This implies also the need and the importance to accurately investigate the possible existence of a family history of the single/combined/generalized frailty syndrome. Consequently, we also suggest creating composite scores considering low bone and muscle mass and quality, low-lean mass, previous history of falls, slow-gait speed, low-grip strength, high-fat mass, dysmetabolic conditions, and levels of physical activity, similar to the existing fracture risk calculators, to boost the effectiveness of rehabilitation programs. This would precociously identify the subject at risk for entering a condition of low-physical activity level, as also the combination of factors to predict adverse musculoskeletal and dysmetabolic outcomes. Therefore, a “multidisciplinary” effort must be made to try obtaining a cumulative frailty score, considering the various existing algorithms to define susceptibility to develop or worsen a frailty condition (e.g., algorithms Framingham, SCORE30, and ASSIGN for the cardiovascular risk; algorithms Diabetes Population Risk Tool and QDiabetes for the risks connected to diabetes; and algorithms FRAX, Qfracture, and Galvan for the fragility fracture risk) (7). Efforts should be aimed at identifying new global, combinate, and risk algorithms enabling the creation of score ranges that allow for a real stratification of cumulative frailty risk. An early, multidisciplinary approach to decrease the global risk of frailty could be implemented, possibly creating appropriate machine learning approaches. Metabolic and dysmobility syndromes may provide an example of how reaching out of our comfort zone of habitual rehabilitation protocols, promoting synergic actions with other specialized medical branches, and creating “Low-physical activity level Units” that aim to go beyond the orthogeriatric units operating already in various national health systems, in a perspective of personalized, person-centered rehabilitation medicine based on the evaluation of individual intrinsic capacity, potentially increasing the overall and cost-benefit effectiveness of our programs (Figure 1). In this wake, the International Classification of Functioning, Disability and Health can be a useful tool to help profiling patients with a low level of physical activity for various conditions and identifying key factors associated with participation in community-based physical activity/rehabilitation.

**Figure 1:**
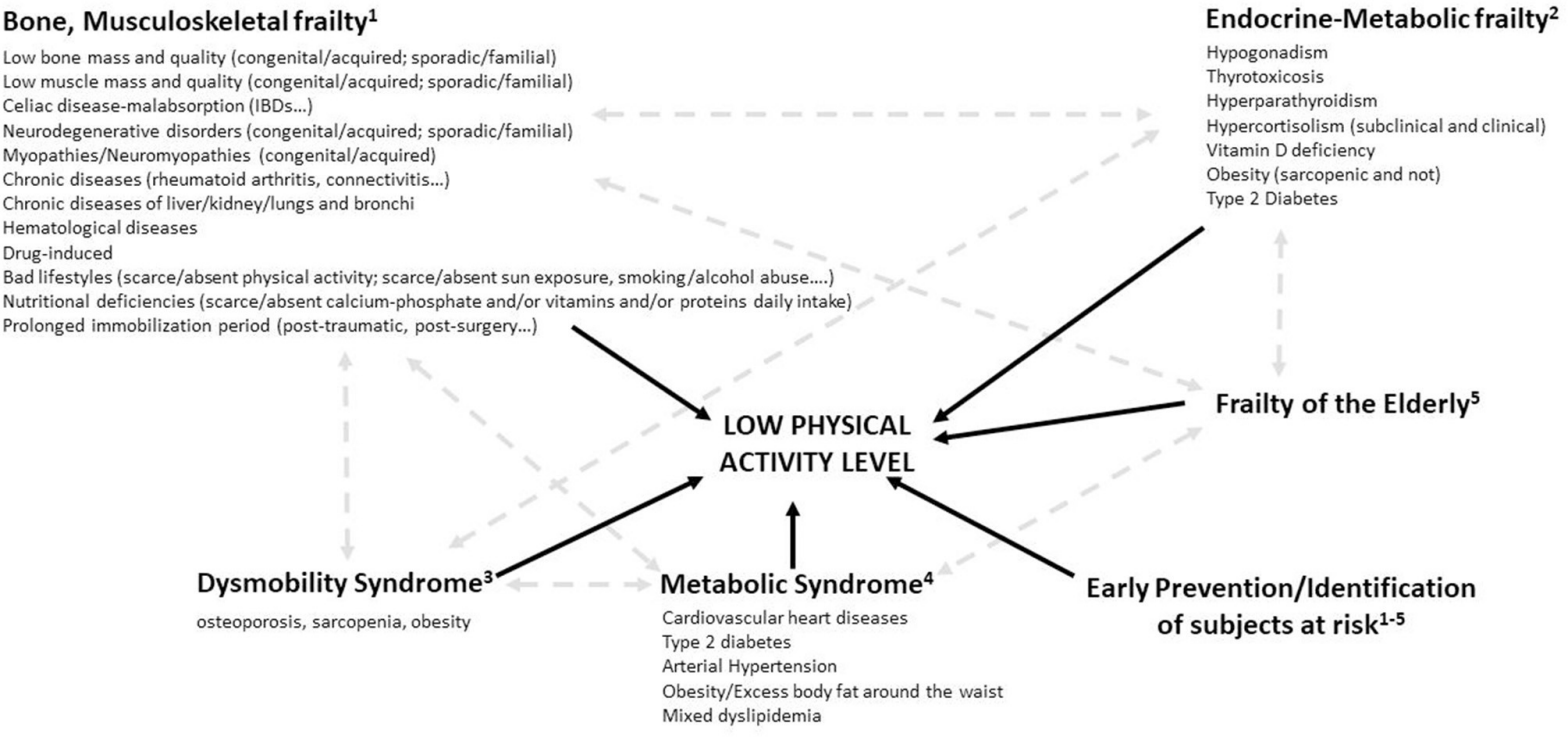
Towards a more comprehensive concept of “global frailty”.

## Author Contributions

AF and PC collaborated in formulating the present opinion paper. Both authors contributed to the article and approved the submitted version.

## Conflict of Interest

The authors declare that the research was conducted in the absence of any commercial or financial relationships that could be construed as a potential conflict of interest. The handling editor declared a past co-authorship with one of the authors PC.

## Publisher's Note

All claims expressed in this article are solely those of the authors and do not necessarily represent those of their affiliated organizations, or those of the publisher, the editors and the reviewers. Any product that may be evaluated in this article, or claim that may be made by its manufacturer, is not guaranteed or endorsed by the publisher.
